# Current approaches in identification and isolation of human renal cell carcinoma cancer stem cells

**DOI:** 10.1186/s13287-015-0177-z

**Published:** 2015-09-16

**Authors:** Mohammed I. Khan, Anna M. Czarnecka, Igor Helbrecht, Ewa Bartnik, Fei Lian, Cezary Szczylik

**Affiliations:** Molecular Oncology Laboratory, Clinic of Oncology, Military Institute of Medicine, ul. Szaserów 128, 04-141 Warsaw, Poland; Institute of Genetics and Biotechnology, Faculty of Biology, University of Warsaw, 02-106 Warsaw, Poland; Institute of Biochemistry and Biophysics, Polish Academy of Sciences, 02-106 Warsaw, Poland; Department of Urology, Emory University School of Medicine, Atlanta, GA 30322 USA

## Abstract

In recent years, cancer stem cells (CSCs)/tumor initiating cells (TICs) have been identified inside different tumors. However, currently used anti-cancer therapies are mostly directed against somatic tumor cells without targeting CSCs/TICs. CSCs/TICs also gain resistance to chemotherapies/radiotherapies. For the development of efficient treatment strategies, choosing the best method for isolation and characterization of CSCs/TICs is still debated among the scientific community. In this review, we summarize recent data concerning isolation techniques for CSCs using magnetic cell sorting and flow cytometry. The review focuses on the strategies for sample preparation during flow cytometric analysis, elaborating biomarkers such as CXCR4, CD105, and CD133. In addition, functional properties characteristic of CSCs/TICs using side population selection through Hoechst 33342 dye, aldehyde dehydrogenase 1, dye-cycle violet, and rhodamine 123 are also discussed. We also include a special focus on enriching CSCs/TICs using three-dimensional cell culture models such as agarose–agarose microbeads and sphere formation.

## Introduction

The identification of adult cancer stem cells (CSCs) in cancerous tissues prompted researchers to understand their role in carcinogenesis. Some cancer cells are more potent than others because of malignancies that arise from either the mutation of normal stem cells or tumor cells that acquire stem cell-like characteristics. CSC theory suggests that these small populations of cells can reproduce and sustain cancer even after subsequent treatment, act more like normal stem cells, and are able to self-renew. These specialized cells are termed CSCs or, more broadly, tumor initiating cells (TICs). Furthermore, research has shown that CSCs/TICs not only exhibit characteristics of normal stem cells, but also gain greater resistance to chemotherapies/radiotherapies. Isolation and further characterization of CSCs/TICs still hold vast mystery among the scientific community owing to a lack of specific stem cell markers. Another difficulty is in determining the methodology employed in isolating CSCs/TICs. In this review, we summarize recent data concerning CSCs/TICs cell isolation techniques and markers for human renal cell carcinoma (RCC).

## Stem cell surface markers in RCC

CD105 is a receptor for transforming growth factor (TGF) located on cell surfaces and takes part in TGF-β signaling by interacting with TGF-β receptors I and/or II. CD105 is important for angiogenesis and is also a prominent marker for mesenchymal stem cells (MSCs) [1]. Bussolati et al. [2] first derived CD105^+^ cells, as TICs, from patient specimens after radical nephrectomy. Magnetically sorted CD105^+^ cells from minced tissue were subjected to further stem cell characterization studies. The frequency of CD105^+^ cells in this study was 8.06 ± 3.3 % and the cells were able to induce tumors in all mice with injected CD105^+^ cells. These results were in agreement with the CSC/TIC hypothesis (Table 1). Moreover, cells with the CD105 marker had much stronger features of CSCs/TICs compared with cells without CD105. The presence of CD105^+^ cells has also been demonstrated in established RCC cell lines 786-O, SMKTR2, SMKTR3, 769-P, Caki-1, Caki-2, ACHN, and RCC-6 [3, 4]. Isolated CD105^+^ cells were further examined for other human MSC markers using the BD Stemflow™ hMSC analysis kit (BD Biosciences, Franklin Lakes, New Jersey, USA). These cells had increased expression of CD73 and CD90 markers and decreased expression of CD44 and CD146. After culturing for 5 days, however, re-analysis of isolated CD105^+^ cells showed that only one-half of the cells were able to maintain the CD105 antigen, suggesting that CD105^+^ cells are highly differentiating and transient in nature [4].

**Table 1:** Comparison of methods for CSC/TIC isolation

Putative markers examined	Isolation method	Cell selection method	Cell lines/specimens examined	Examined CSC/TIC criteria	Reference
ABCG2, CD90, CD105, CD133, EpCAM	ALDH1	Flow cytometry	Renal: ACHN, KRC/Y	Sphere formation assay, drug sensitivity assay, in-vivo tumorigenicity	Ueda et al., 2013 [45]
Not examined	ALDH1	Flow cytometry	Ovary: AMOC-2, HUOA, OVCAR-3, ES-2, RMG-1, TOV-21G	Sphere formation assay, in-vivo tumorigenicity, invasion assay	Kuroda et al., 2013 [62]
ABCB1, ABCG2, ABCC1	Hoechst 33342	Flow cytometry	Renal: 769P, 786-O, OS-RC-2, SN12C, SKRC39	Drug sensitivity assay, clone formation, in-vivo tumorigenicity	Huang et al., 2013 [47]
Sox-2, POU5F1, Cdh1, Cdh2, Snai1, Snai2, Twist1, Twist2	Hoechst 33342	Flow cytometry	Renal: RENCA, ACHN, CAKI-1, SMKT-R2, SMKT-R3	In-vivo tumorigenicity	Nishizawa et al., 2012 [23]
CD3, CD4, CD8, CD24, CD44	Rhodamine	Flow cytometry	Renal: 786-O	Colony formation assay, radiation sensitivity assay, in-vivo tumorigenicity	Lu et al., 2013 [21]
CD24, CD44, CD31, CD146, CD90, CD73, CD29, CK7, CD133, Vimentin, Musashi, Nanog, Pax2, Oct-4	CD105-based cell selection	Magnetic-activated cell sorting	Renal carcinoma specimens	Sphere formation assay, in-vivo tumorigenicity	Bussolati et al., 2008 [2]
CD34, CD45, CD14, CD44, CD29, CD73, CD105, Pax2, CD117, Cytokeratin, Vimentin, E-cadherin	CD133-based cell selection	Magnetic-activated cell sorting	Renal carcinoma specimens	In-vivo tumorigenicity	Bruno et al., 2006 [8]
CD24, CD29, CD44, CD73, CD146, CXCR1, CD34, CD90, CD105, CD133, ALDH1, CD117, CXCR4, Nanog, POU5F1, Sox-2, Cytokeratin, Vimentin	CXCR4-based cell selection	Flow cytometry	Renal: RCC-26, RCC-53, SK-RC-17	In-vivo tumorigenicity, sphere formation assay, drug sensitivity assay	Gassenmaier et al., 2013 [14]
CD44, CD24, Sca1, Oct-4, Wnt, ABC white 2, β-Catenin,	Encapsulation of tumor colony	Cell recovery from 3D culture inside macrobeads	Renal: RENCA	Not examined	Smith et al., 2011 [66]
			Breast: MMT, K12, MCF7		
			Uterus: JEG-3		
			Prostate: DU145		
			Colon: HCT116		
			Bladder: J82		
Oct-4	Encapsulation of tumor colony	Cell recovery from 3D culture inside macrobeads	Renal (mice): RENCA	Chemotherapy sensitivity assay, in-vivo tumorigenicity	Gazda et al., 2013 [84]
CD133, CD34, CD24, CD14, CD105, CD45, Oct-3/4, Nanog, Sox-2, E-cadherin, VEGF R2/KDR/Flk1, HGC	CTR2^+^/CD133^+^/CD24^+^ cell selection	Flow cytometry	ccRCC specimens	In-vivo tumorigenicity, chemotherapy sensitivity assay, cell cloning and differentiation assay	Galleggiante et al., 2014 [9]
Oct-3/4, Nanog, Pax-2	ALDH1	Flow cytometry	Renal carcinoma specimens	In-vivo tumorigenicity, sphere formation assay, drug sensitivity assay	Wang et al., 2015 [63]
			Renal: ACHN, CAKI-2		

*ALDH* aldehyde dehydrogenase, *ccRCC* clear cell renal cell carcinoma, *CSC* cancer stem cell, *TIC* tumor initiating cell, *VEGF* vascular endothelial growth factor

CD133, also called Prominin-1 or AC133, is a pentaspan transmembrane protein first identified in mouse neuroepithelial stem cells and later described in human hematopoietic stem cells [5, 6]. The CD133^+^ cell population has been identified as resident renal progenitor cells in adult normal human kidney [7] and contributes to tumor vascularization and angiogenesis. Bruno et al. demonstrated a contributory role of CD133^+^ progenitor cells derived from human RCC in tumor vascularization [8]. CD133^+^ and CD133^−^ cells were magnetically sorted using the magnetic-activated cell sorting (MACS) system to evaluate in-vivo angiogenesis and tumorigenic potential. CD133^+^ or CD133^−^ cells were transplanted into SCID mice with or without cells from the K1 RCC cell line at different ratios (i.e., 1:100 for CD133^+^/K1 cells, 100:1 for CD133^+^/K1 cells). Results were compared with mice injected with K1 cells alone (1 × 10^4^ to 1 × 10^6^ cells). Injected CD133^+^ cells alone did not form tumor after 6 months. However, CD133^+^ cells cotransplanted with the RCC cell line K1 significantly enhanced tumor growth and development. Moreover, newly formed vessels within the tumor were positive for both human HLA class I and human CD31, confirming its human origin. The fact that tumor vessels were derived from differentiating CD133^+^ progenitor cells plus K1 cells was later confirmed by fluorescence in-situ hybridization for expression of human chromosome X [8]. Others have identified CD133^+^ cells that coexpressed CD24 and CTR2 antigens from RCC patients [9]. CD133^+^/CD24^+^/CTR2^+^ cells were more tumorigenic and have more potential to behave as CSCs/TICs compared with cells which do not express these markers.

CXCR4 chemokine receptor belongs to the superfamily of G protein-coupled receptors and has been found to be a prognostic marker in various types of human cancer. The chemokine CXCL12 (SDF-1) acts as a chemoattractant to the CXCR4 receptor-positive primary tumor cells, and drives them toward secondary metastatic sites. CXCL12–CXCR4 axis signaling is known to play a pivotal role in the homing of normal stem cells [10]. Interestingly, CSCs have been found to express CXCR4 receptor, and this CXCL12–CXCR4 axis is also involved in trafficking/metastasis of CSCs to the organs which are highly rich in CXCL12, such as the lymph nodes, lungs, liver, and bones [11, 12]. Schrader et al. analyzed the CXCL12/CXCR4 expression and function in four human RCC cell lines: A-498, Caki-1, Caki-2, and HA-7 [13]. Cell surface expression analysis of CXCR4 antigen using fluorescence-activated cell sorting (FACS) showed that only Caki-1 and A-498 cell lines expressed this antigen, which was also confirmed through RT-PCR. Another breakthrough study [14] identified the pivotal role of CXCR4 receptor in maintaining cancer stem-like features and metastasis particularly in relation to RCC. The study examined two RCC lines, RCC-26 and RCC-53, derived from primary tumors of clear cell RCC (ccRCC) patients with disease stage I and IV, respectively. These differ significantly in their capability of forming tumor spheres. Under nonadherent conditions, RCC-56 cells form numerous spheres compared with RCC-26 cells, which form rather poor spheres. Flow cytometry analysis after dissociation of spheres demonstrated an increased fraction of CXCR^+^ cells in a prosphere culture condition compared with a monolayer culture environment. In addition, sorted CXCR^+^ cells from both cell lines have increased expression for stemness genes, such as *Nanog*, *Oct-3/4*, and *Sox-2*, and higher resistance to tyrosine kinase inhibitors, such as sunitinib, sorafenib, and pazapanib [14].

CD44 is a single-chain transmembrane glycoprotein that has a major role as an adhesion molecule for the extracellular matrix which binds primarily to the extracellular glycosaminoglycan hyaluronan [15, 16]. CD44, a cell surface antigen, has been implicated in a wide variety of physiologic processes including wound healing, cell growth, survival, and differentiation as well as tumor cell migration, invasion, and metastasis. Some have also reported CD44 as CSCs/TICs [16, 17], although this nature is still controversial [18] (Table 2). Furthermore, clinical data have also suggested positive correlation between CD44 expression and metastasis [19]. Debeb et al. [20] described several features of CD44^+^/CD24^−^ cells as CSCs/TICs which were derived from the human embryonic kidney cell line 293T. In-vivo observation of serially transplanted 293T cells showed self-renewal and sphere formation when cultured in a stem cell-promoting suspension culture. Moreover, CD44^+^/CD24^−^ cells showed increased aldehyde dehydrogenase (ALDH) activity in three-dimensional culture compared with two-dimensional cell culture conditions. Furthermore, the three-dimensional spheres from CD44^+^CD24^−^ cells were resistant to chemotherapy and radiotherapy [20]. These findings suggest the importance of CD44^+^CD24^−^ cells as CSCs/TICs, but the underlying mechanisms are unclear. Similar results were also reported in Rh123^high^ sorted cells and spheres from ccRCC cell lines 786-O, ACHN, and Caki-1 [21, 22] and in CD105^+^ cells isolated from RCC specimens [2].

**Table 2:** Marker-based RCC CSC phenotypes

Marker (positive)	Sox, Oct, Nanog, Bmi-1	EMT markers^a^	Spheres	Mice	Cell line	RCC type	Reference
CD44, CD49f, ALDH1, CD24↓	+	E↓, M↑+/−	–, ROS	+	Caki-1	ccRCC	Mahalingaiah et al., 2015 [17]
CD24, CD44	+	M↑	+	–	ACHN, Caki-1	pRCC, ccRCC	Lichner et al., 2014 [22]
–	+	E↓, M↑	+, TNFα	–	ACHN, 786-0	pRCC, ccRCC	Zhang et al., 2014 [27]
DCLK1↑ ALDH1	+	M↑	–	–	Caki-2	pRCC	Weygant et al., 2015 [29]
CD44, CD133, CXCR4	NS, B-cathenin	M↑	+, PIK3R1↓	–	786-0, A-704	ccRCC, RCC	Lin et al., 2015 [30]
ALDH1	+	–	–	+	Caki-2, ACHN	pRCC, pRCC	Wang et al., 2015 [63]
CXCR4, CD105↓, CD133(−)^b^	+	–	+	+	RCC-26, RCC-53	RCC	Gassenmaier et al., 2013 [14]
CD24, CD44, CD133(−), CD105(−)	+	B-c↑	+	+	SK-RC-42	RCC	Zhong et al., 2010 [72]
CD24, CD44	Side population	–	+	+	786-0	ccRCC	Lu et al., 2013 [21]
ALDH1	–	NS	+	+	ACHN	pRCC	Ueda et al., 2013 [45]

^a^Mesenchymal (M): vimentin, N-cadherin, fibronectin, Snail, Fox-2, Slug, ZEB1. Epithelial (E): E-cadherin, cytokeratin, B-catenin

^b^CD24, CD29, CD44, CD73, CD146—highly expressed on cell line = not considered as CSC markers

*↓* decrease, *↑* increase, *ALDH* aldehyde dehydrogenase, *B-c* B-catenin, *ccRCC* clear cell renal cell carcinoma, *CSC* cancer stem cell, *EMT* epithelial–mesenchymal transition, *NS* not specified, *pRCC* papillary renal cell carcinoma, *RCC* renal cell carcinoma, *ROS* reactive oxygen species, *TNFα* tumor necrosis factor alpha

## Intracellular markers in RCC

Heat shock protein (HSP) 40 family member DnaJ (Hsp40) homolog, subfamily B, member 8 (DNAJB8) has an important role in maintaining the CIC/TIC phenotype in RCC and colon cancer [23, 24]. Overexpression of DNAJB8 enhances the expression of stem cell markers and tumorigenicity. The human RCC cell lines ACHN, Caki-1, SMKTR2, SMKTR3, and HEK293T together with murine RENCA cells and BALB/3 T3 cells from murine fibroblasts were analyzed for expression of DNAJB8 [23]. The ratio of side population (SP) cells derived from ACHN and RENCA cells was 2.6 % and 18 %, respectively, using Hoechst 33342 dye staining. RT-PCR analysis of these isolated SP cells showed that *DNAJB8* was predominantly expressed together with *Sox-2* and *POU5F1* genes. Western blotting and immunostaining using SP cells also correspond with preferential expression of DNAJB8 protein, confirming stem cell-like phenotypes [23]*.*

Micro RNAs (miRNAs) are small, noncoding, single-stranded RNA molecules that act as posttranscriptional regulators. They are required for the maintenance of normal pluripotent embryonic stem cells in mice [25]. The role of miRNAs has been documented in many cancers, including breast cancer, glioblastoma, and prostate cancer [26]. In RCC, Lichner et al. [22] examined the influence of miR-17 on cancer spheres with cancer stem-like properties from two metastatic RCC cell lines: ACHN and Caki-1. Spheres were obtained from ACHN and Caki-1 cells while cultured in serum-free defined media (SFDM) supplemented with fibroblast growth factor (FGF), epidermal growth factor (EGF), and B27. Sphere-forming cells were later examined for CD24 and CD44 CSC markers. Spheres from Caki-1 and ACHN cell lines were positive for CD24^+^/CD44^+^ at 10 % and 9.37 %, respectively. In addition, spheres from both cell lines exhibited greater tumorigenic ability and increased expression for stem cell and mesenchymal markers in vivo. However, miR-17 was significantly downregulated in both cell lines. Transfection with anti-miR-17 led to a rapid formation of three-dimensional spheres but did not affect the sphere-forming efficiency. Interestingly, miR-17 inhibition resulted in 2.4-fold and 1.96-fold increase in the number of colonies formed in Caki-1 and ACHN, respectively, demonstrating capabilities of inducing self-renewal and significantly increased expression for mesenchymal markers such as ZEB1, ZEB2, vimentin, and N-cadherin and for cancer stem markers CD24 and CD44 [22].

Emerging evidence suggests that acquisition of epithelial–mesenchymal transition (EMT) is associated with tumor invasion and metastasis. EMT plays an important role in tumor progression via an acquired ability to self-renew, spread, and recur. Together, these features of EMT strongly suggest a possible relationship with the CSC phenotype. In a recent study, the EMT process was artificially induced inside the cell culture of RCC cell lines ACHN and 786-O to enrich their stemness features [27]. ACHN and 786-O cells were treated with 50 ng/ml tumor necrosis factor alpha for 7 and 14 days, respectively. This led to a loss of epithelial morphology and acquired mesenchymal appearance, and increased expression of mesenchymal protein markers such as Vimentin, Slug, and ZEB1. In addition, these EMT signature RCC cells had upregulation of stemness genes such as Oct-4, Nanog, and Bmi-1, together with increased potential of tumor sphere formation [27].

In contrast to artificially induced EMT, Li et al. [28] applied a different approach which can reverse the EMT process and inhibit CSC-like characteristics in RCC. Honokiol extracts isolated from Magnolia spp. bark can suppress the proliferation of RCC cells in vitro. Furthermore, there can be an increase in protein expression of epithelial markers E-cadherin and decrease in expression of mesenchymal markers such as fibronectin and Vimentin. Honokiol significantly decreases the number and size of tumor sphere formation and decreases the number of SP cells. All of these findings suggest that honokiol regulates EMT and CSC/TIC properties by modulation of miR-141 and its target gene ZEB2 [28].

Analysis of The Cancer Genome Atlas’ Kidney Renal Clear Cell Carcinoma (TCGA KIRC) dataset revealed that Doublecontin-like kinase 1 (DCLK1) is epigenetically overexpressed in RCC tumors regardless of the disease stage [29]. Recently, Weygant et al. [29] investigated the importance of DCLK1 in regulation of EMT and maintaining stemness features. Silencing of the DCLK1 gene using DCKL1 small interfering RNA (siRNA) in primary RCC-caki-2 cells resulted in decreased expression of EMT transcriptional factors (SNAI1, SNAI2, TWIST1, ZEB1, and Vimentin). Moreover, this gene silencing also led to reduced expression of stemness and pluripotency factors MYC, Nanog, Oct-4, Sox-2, and ALDH1A1. These results illustrate the vital role of DCLK1 knockdown in reducing the invasive and metastatic capability of RCC. High protein expression of PIK3R1 has been observed in normal kidney tissues. Recent findings showed that PIK3R1 expression correlated with RCC progression and metastasis [30]. Functional study of PIK3R1 knockout revealed its significant role in RCC cell migration and proliferation [30]. Moreover, knockout PIK3R1 cells displayed a mesenchymal morphology and increased expression for EMT-related factors in vitro.

## Multiple marker phenotype of RCC CSCs

Coexpression of multiple putative stem markers in identifying CSCs has been studied in many cancers [31, 32]. Recently, Galleggiante et al. [9] identified cancer stem-like cells using the multiple marker CTR2^+^/CD133^+^/CD24^+^ from patients with clear RCC. This resident subpopulation showed stemness properties similar to tubular adult renal progenitor cells (tAPCs) derived from healthy kidney. CD133^+^/CD24^+^ cells isolated from tumor kidney tissue were more undifferentiated than tAPCs. CTR2 is localized on the cell surface membrane and coexpressed with CD133^+^/CD24^+^ cells. Expression of CTR2 has been reported by others in human RCC. Coexpression of CTR2 with CD133/CD24 protein could be useful in discriminating between CSCs and the normal renal cell population [9]. Moreover, in RCC patients a significant role of CTR2 in cisplatin-based resistance also been reported. Another example of a coexpression approach is to identify CSCs in RCC using CD133/CXCR4-based cell selection. Resistance to sunitinib is a major obstacle in RCC treatment [33]. Varna et al. [34] studied the role of CD133^+^/CXCR4^+^ cells in the course of developing such resistance. RCC specimens obtained from patients before and after sunitinib treatment were analyzed for cells expressing CD133. CD133-expressing cells were significantly more numerous in sunitinib-treated patients than in untreated patients. Interestingly, CD133^+^ cells coexpressed CXCR4, which showed higher tumorigenic potential in vitro [34].

## Methods for the isolation of cancer stem-like cells

### Cluster of differentiation identification

Another well-known method for stem cell separation is MACS. This widely used method isolates different types of cells including human lymphocytes, dendritic cells, mega-karyotic cells, and granulocytes [35–38]. The use of small magnetic beads conjugated with antibodies allows for direct enrichment and isolation of cells without further staining. Bussolati et al. [2] used this method to isolate tumor-initiating cells in specimens from RCC obtained after radical nephrectomy. Specimens were minced and digested using collagenase II. They used CD105 (endoglin) antibody conjugated with magnetic beads that recognized surface antigens for CD105^+^ cells. Before isolation, 2 × 10^7^ cells were initially labeled using 80 μl monoclonal anti-CD105 antibody coupled with magnetic beads in cold MACS running buffer (phosphate-buffered saline (PBS) without Ca^2+^ and Mg^2+^ supplemented with 1 % bovine serum albumin (BSA) and 5 mM ethylenediamine tetraacetic acid (EDTA)) for 20 min. Later, cells were washed twice using MACS buffer and were suspended in MACS buffer. The cell suspension was passed through a MACS magnetic column held on the MACs separator stand. During this process, CD105^+^-labeled cells are collected inside the MACS column and can be further collected by pushing a plunger into the column [2].

### Side population isolation

One of the most popular and commonly used methods to identify CSCs/TICs is SP cell selection using Hoechst 33342 dye [39–41]. The SP phenotype is a manifestation of primitive cells’ ability to efficiently efflux the fluorescent DNA-staining dye (i.e., Hoechst 33342) and can be used to isolate these cells using flow cytometry [42]. This is in contrast to the protein marker approach for isolation, where cells are first immunostained with fluorescently conjugated antibodies. Later, positively fluorescent labeled cells are sorted using flow cytometry whereas the Hoechst 33342 dye efflux assay isolates cells based on the ability of stem cells to actively efflux cytotoxic agents like Hoechst 33342, a bis-benzimidazole that binds to adenine–thymine-rich regions of the minor groove of DNA. SP cells have been analyzed by this technique from healthy hematopoietic stem cells inside bone marrow [43]. Moreover, this study showed that the SP also has exceptionally strong features of stem cell-like activities. There are many reports that suggest a role for CSCs/TICs in solid tumors, but only few reports contribute toward involvement of SP cells in RCC [23, 44–46].

Hoechst 33342 DNA binding dye (SIGMA-Aldrich, Saint Louis, USA) was used in two RCC cell lines derived from primary lesions of Japanese females (KRC/Y) and from malignant (ACHN) pleural effusion from Caucasian males with metastatic RCC [45]. Cells were cultured in monolayers until they reached nearly 80 % confluence, and then cells were harvested using accutase and suspended at a density of 1 × 10^6^/ml in PBS with 2 % fetal bovine serum (FBS). Suspended cells were incubated with Hoechst 33342 dye at 37 °C for 60 min. The samples were washed, centrifuged, and resuspended in 2 ml cold PBS with 2 % FBS. Propidium iodide (PI) was then added at a concentration of 1 mg/ml to measure dead cells. Cells were filtered through a 40 μm membrane. Finally, the SP cell analysis was carried out through a FACS AriaII flow cytometer (BD Biosciences, San Jose, CA, USA). The SP percentage in ACHN and KRC/Y cells was 1.4 % and 1.7 %, respectively, whereas upon treating cells with reserpine no SP cells were observed. Hoechst 33342 dye staining was used for analysis of SP cells and non-SP cells in five established human RCC cell lines: 769P, 786-O, OS-RC-2, SN12C, and SKRC39 [47]. This method proved effective only in the 769P cell line; in the other cell lines, the ratio of SP cells was either too low or nondetectable. In addition, the expression of the ATP-binding cassette (ABC) transporter ABCB1, a member of the MDR/TAP protein subfamily, was higher in SP cells compared with non-SP cells, which was further confirmed by RT-PCR and western blot [47].

The ability to recognize Hoechst-positive SP cells depends on the differential efflux of cells by multidrug-like transporter protein. Great attention is therefore required for Hoechst concentration, staining time, and staining temperature. All steps are critical before analysis. In the RCC cell line 769-P, SP analysis and cell isolation was performed with a small modification as described previously by Goodell et al. [43]. The cells were mobilized using trypsin instead of accutase, and were suspended in prewarmed RPMI-1640 containing 2 % FBS and 10 mmol/l HEPES [23]. RCC cells were spun down and resuspended at 1 × 10^6^ cells/ml in RPMI-1640, followed by standard incubation with 5 mg/ml Hoechst 33342 dye at 37 °C for 90 min in the dark. After staining, cells were spun down and resuspended in cold HBSS again containing 2 % FBS and 10 mmol/l HEPES for flow cytometry analysis. Another option is to suspend cells in prewarmed Dulbecco’s modified Eagle’s medium (DMEM) with 5 % FBS. Hoechst 33342 dye can be added at a final concentration of 2.5 mg/ml in the presence or absence of verapamil (50 mmol/l). The cells may then be incubated at 37 °C for 90 min with intermittent blending [23].

Hughes et al. [48] also employed the Hoechst SP approach for stem cell identification from renal epithelial cells and a novel method to further characterize SP cells using synchrotron radiation–Fourier transform infrared (SR-FTIR) spectroscopy. In this method, renal epithelial cells were first stained with Hoechst 33342 dye and sorted using a FACS vantage cytometer to define the SP gate. Thereafter, SR-FTIR spectroscopy was used to acquire single-point spectra and biochemical maps for each cell type. SP sorted cells were very small, consisting of a nucleus and limited cytoplasm, demonstrating that these cells have a distinct chemical phenotype compared with the remaining renal cells [48].

Another CSC identification method is based on the cells’ ability to efflux toxins using rhodamine 123 (Rh123), which preferentially accumulates in active mitochondria. The content of Rh123 dye in the cells helps to isolate cells with progenitor characteristics [49, 50]. Rh123 is a cell-permeable fluorescent dye that stains mitochondria in cells, since there is a correlation between the amount of ATP in cells, the fluorescence intensity of Rh123 dye, and drug resistance though the ABC [51, 52]. In the human RCC line 786-O, two different fractions of cells were observed: Rh123^high^ cells and Rh123^low^ cells. The 786-O cell line was first grown in RPMI-1640 media supplemented with 10 % heat-inactivated FBS using normal incubation conditions [21]. Once the cells reached a logarithmic growth phase, they were harvested using 0.25 % trypsin. Cells were washed twice with calcium/magnesium-free PBS and resuspended in ice-cold RPMI-1640 with 5 % FBS at a concentration of 1 × 10^6^ cells/ml and incubated for 10 min at 37 °C in 5 % CO_2_. Rh123 fluorescent dye was added at a concentration of 10 μg/ml and incubated for 20 min in the dark. Cells were washed twice with PBS and examined by flow cytometry. Sorted cells were divided into two groups: rhodamine high active cells (Rh123^high^) and rhodamine low active cells (Rh123^low^). Rh123^high^ has been further assessed for other stem cell-like properties, such as colony formation, radio-sensitivity, screening for stem cell markers (CD24 and CD44), and tumor formation in SCID mice. The human primary RCC line 786-O was used to isolate the CSC/TIC population based on the Rh123 fluorescent intensity [21]. The cells with increasing intensity for Rh123 dye exhibit characteristics of stem-like cells that include higher colony formation capability, higher differentiation potential, and resistance to radiation and tumor formation in NOD/SCID mice [21].

CSCs/TICs are hypothesized to be resistant from toxins, hypoxia, and radiation [53, 54]. One possible mechanism behind toxin efflux is through the expression of ABC transporter proteins. Moreover, protein overexpression of members of the ABC transporter superfamily (ABCB1, ABCC1, and ABCG2) contributes to drug resistance and exhibits the SP phenotype. Hence, another method to isolate CSCs/TICs is based on the ability to efflux toxins using the dye-cycle violet (DCV) cell staining procedure. Recent studies have shown efficacy of this membrane-permeable dye in identifying the SP in bone marrow and cultured cell lines of prostate cancer [51, 55, 56]. However, the role of DCV in CSC identification in RCC is still untested and not enough data have been reported. DVC is an alternative to Hoechst 33342 dye staining and is also a substrate for the ABCG2 efflux transporter protein that can be excited by a violet laser. Samples were prepared in suspension and Fumitremorgin C (FTC) was used to inhibit ABCG2 function to facilitate localization of DVC effluxing cells in prostate cancer [56, 57]. In darkness, 1 μl DVC was added to 1 × 10^6^ cells in 0.5 ml epithelial culture media with or without 0.5 μl FTC [56]. Sample tubes were vortexed and incubated at 37 °C for 20 min. After 20 min, samples were vortexed and 1 μl DVC was added again to obtain a final concentration of 10 μM. Samples were again incubated at 37 °C for 90 min and vortexed every 15–20 min during incubation. Later, samples were centrifuged at 800 × *g* for 5 min at 4 °C and washed with 1× PBS before resuspension in 0.5 ml Hanks solution with 5 % FBS buffer. Finally, 5 μl of 7-aminoactinomycin D (7AAD) was added to the samples in the dark. Cells were sorted using FACS [56].

### Aldehyde dehydrogenase identification

ALDH1 is an enzymatic approach for both normal stem cell and CSC/TIC identification. Researchers have used the ALDEFLUOR assay where high expression of ALDH1 enzyme activity corresponds to CSC/TIC markers in different types of cancer, including lung cancer, prostate cancer, breast cancer, bladder cancer, liver cancer, ovarian cancer, and malignant melanoma [58–63]. Ueda et al. [45] researched the biological features of ALDH1^+^ and ALDH1^−^ in samples prepared from SP and non-SP cells, drug-treated cells, and cells cultured under hypoxic conditions previously isolated from two RCC cell lines. SP and non-SP cells were cultured for 72 h. Later, samples were suspended in ALDEFLUOR assay buffer containing ALDH substrate with 50 mg dry bodipy-aminoacetaldehyde, with or without 5 μl ALDH inhibitor (diethylaminobenzaldehyde) as a negative control. Samples were incubated for 60 min at 37 °C inside the incubator prior to analysis using flow cytometry. An almost twofold increase of ALDH1^+^ cells was observed in the metastatic ACHN cell line (15.3 %) compared with the primary KRC/Y cell line (6.5 %). This research demonstrated that the number of ALDH1^+^ SP cells (32.7 %) was double that of non-SP cells (14.6 %). In addition, the sphere-forming ability of ALDH^+^ cells was higher in both RCC cell lines compared with ALDH^−^ cells and RCC cells were capable of forming tumors in mice [45].

### Three-dimensional cell culture

The three-dimensional culturing of tumor cells can be used to closely mimic the in-vivo tumor microenvironment, unlike the traditional two-dimensional monolayer culture [64]. This novel three-dimensional culturing model for tumor cells in polymeric scaffolds was first discussed by Jain et al. [65]. In RCC, this method was employed to enrich the cancer stem-like cells from the mouse renal adenocarcinoma RENCA cell line using macrobeads [66]. RENCA cells were grown for 5 days inside six-well plates at a density of 15,000 cells per well containing 10 % neonatal calf serum (NCS) in RPMI-1640 media. Separately, 100 μl of 0.8 % low-viscosity agarose was prepared in minimum essential medium (MEM), and kept at 51–53 °C followed by mixing with 1.5 × 10^5^ RENCA cells. The agarose cell suspension was expelled into sterile mineral oil at room temperature to form a smooth, semi-solid core of macrobeads. Mineral oils were removed with RPMI-1640 and the core was cultured overnight at 37 °C under 5 % CO_2_. The following day, the core was rolled in a sterile spoon containing 1 ml of 4.5 % agarose in MEM maintained at 61–63 °C to apply an outer coating of agarose. These agarose–agarose macrobeads were transferred to mineral oil to form smooth macrobeads and washed again with RPMI-1640 prior to culture. Macrobeads were cultured at 37 °C in 5 % CO_2_ in a 90-mm Petri dish containing 10 macrobeads in 40 ml of 10 % NCS-supplemented RPMI-1640 media. Immunofluorescence studies on RENCA macrobeads show enrichment of CSC/progenitor cell-like attributes [66]. These cells tested positive for CD44 (CD24^−^), stem cell antigen-1 (SCA-1), and OCT4 protein.

Culturing conditions for CD105^+^ clones to grow as a floating tumor sphere were first modified by Bussolati et al. [2]. Cells were plated at 1 × 10^3^ cells/ml in serum-free DMEM/F12 with 10 ng/ml basic FGF and 20 ng/ml EGF together with 5 μg/ml insulin and 0.4 % BSA. Spheres were further dissociated using nonenzymatic solution every 7–10 days to evaluate signal cell sphere formation. Gassenmaier et al. [14] obtained tumor spheres from two RCC cell lines derived from primary tumors of ccRCC patients with disease stage I (RCC-26) and stage IV (RCC-53). Tumor spheres were formed in serum-free medium consisting of DMEM/F12, 1 % insulin–transferrin–selenium-X, 2 % B27, 20 ng/ml EGF, and 20 ng/ml basic FGF. Cells were seeded in low attachment plates and grown for up to 7 days. The number of spheres was assessed after 4 days to avoid miscalculation connected with aggregates of spheres [14].

Modification of the cell culture technique to obtain CD105^+^ cells from floating tumor spheres has been demonstrated by Hu et al. [67]. In their method, cultures were disrupted into single cells using a 37 μm filter, and cultured in 96-well plates with a low adhesion surface. The cells were added to each well at a concentration of 300–500 cells/well. Cultures were fed with serum-free medium supplemented with N2 (1:100), heparin (5 g/ml), EGF and basic FGF (both 20 ng/ml), insulin (20 ng/ml), B27 (1 %), and human leukemia inhibitory factor (100 ng/ml) [67].

Spheres were obtained from ACHN and Caki-1 cells while culturing in SFDM supplemented with FGF, EGF, and B27. Spheres from those Caki-1 and ACHN cell lines were positive for CD24^+^/CD44^+^ at 10 % and 9.37 %, respectively [22]. In addition, spheres from both cell lines exhibited greater tumorigenic ability and increased expression for stem cell and mesenchymal markers in in-vivo studies, demonstrating capabilities of inducing self-renewal and significantly increased expression for mesenchymal markers such as ZEB1, ZEB2, vimentin, and N-cadherin, and cancer stem markers CD24 and CD44 [22].

### Serum-free culture-based approach

In 1992, Reynolds and Weiss [68] developed the sphere culture method with cells isolated from adult mammalian brain. This method has since been employed to isolate cells with CSC/TIC characteristics. Many studies confirmed that under serum-free conditions and in the presence of specific mitogens, such as EGF and basic FGF, the CSC/TIC population can be enriched [69–71]. The human RCC cell line SK-RC-42 was cultured by employing the sphere culture method for enriching CSCs/TICs [72]. Initially, SK-RC-42 cells were maintained as a monolayer in DMEM/F12 with 10 % FBS. Tumor spheres were cultured by growing cells as a monolayer in serum-free DMEM/F12 with 20 ng/ml EGF, 20 ng/ml basic FGF, and B27 supplements. After 7–10 days, the tumor sphere was collected using gravity and was dissociated using 0.1 % trypsin and 1 mM EDTA. The cells were passed through 40 μm nylon mesh and plated at 1000 cells/ml in serum-free DMEM/F12 as described earlier to produce clonal spheres. These spheres were further tested for putative stem cell markers such as CD24, CD44, CD105, and CD133. However, expression of CD105 was observed in all cultures, which corresponds with the previous finding by Bussolati et al. [2] showing CD105^+^ cells as TICs. In contrast, Zhang et al. [27] used the serum-free approach to assess the tumor sphere-forming ability after artificially inducing EMT in RCC cell culture rather than enriching the stemness properties in cell culture.

## Conclusions

Multiparametric flow cytometry allows simultaneous analysis of different cellular features with high performance and reliability. This method can be used to separate cells of interest using expression markers or functional properties through FACS [73]. However, isolation and analysis of tissue samples with this method can cause artifacts that bias analysis [74]. New putative CSC/TIC markers on the basis of surface expression in RCC cells are under investigation. However, the reliability of CSC/TIC markers in cancer research is still under debate [75]. CSCs/TICs accumulated mutations in some of the key signaling pathways, such as Wnt, Notch, and Hedgehog, that are mainly responsible for proliferation, apoptosis, cell cycle, repair, and other functional features [76]. These genetic mutations trigger the tumor phenotype in normal stem cells. The cellular phenotype of CSCs/TICs is transient and may vary during the development and growth of in-vivo and in-vitro tumor culture. Phenotypic changes that occur in in-vitro experimental conditions also influence marker expression analysis and detectability [77]. It has often been observed that some marker proteins on the cell surface are sensitive to enzymatic digestion, particularly when cells need to be digested before flow cytometry analysis [78]. Optimization of culturing conditions is therefore necessary before isolation and flow cytometry analysis. The choice of protease for cell detachment is highly important for preserving the antigenicity of individual cell surface markers for the identification and sorting of CSCs/TICs [79]. An alternative to trypsin is the use of Accutase, which is often viewed as being gentler for cellular detachment without the need for a neutralizing solution.

CSCs/TICs often express ABC transporter proteins, which enables use of DNA dyes such as Hoechst 33342 and Rh123 and the use of flow cytometry to identify them as a “side population” [80]. When SP cells are identified though staining, it is not clear whether they represent the entire pool of CSCs in a tumor or just part of it [73]. Differences in staining protocols may contribute to such discrepancies as the cell staining incubation time, dye concentrations, cell densities, and different gating strategies [81, 82]. Cell line-specific properties also influence analysis [82]. Proper measures should therefore be taken before adjusting the staining protocol.

There are variations that exist in the Hoechst 33342 staining protocol, and these variations therefore have to be taken into consideration before interpreting data [81]. Different manufacturers offer variation in the final working concentration of Hoechst 33342 dye. Commonly, the final working concentration varies from 0.1 to 10 μg/ml Hoechst 33342 dye in a reaction tube. The correlation between the incubation time and the Hoechst dye concentration is crucial for dye uptake kinetics in characterizing CSCs [83]. These variations in protocol probably lead to skepticism and uncertainty about the accuracy of the Hoechst method for stem cell detection [73].

Analysis of stem cell features though the SP, ALDH, or cell surface markers exclusively have limitations to identify CSCs/TICs in RCC. For instance, Bussolati et al. [2] analyzed the cell surface marker CD105 alone as CSCs/TICs in RCC tissues. On the other hand, SP analysis alone was sufficient to identify CSC/TIC phenotypes in the RCC cell line 769-P [47]. Combining several markers is therefore suggested. A combination of SP or ALDH analysis with cell surface markers such as CD105, CD133, or CXCR4 in investigating CSCs/TICs in RCC will provide significant and valuable data in the future.

## Abbreviations

7-AAD: 7-Aminoactinomycin D
ABC: ATP-binding cassette
ALDH: Aldehyde dehydrogenase
BSA: Bovine serum albumin
ccRCC: Clear cell renal cell carcinoma
CSC: Cancer stem cell
DCV: Dye-cycle violet
DMEM: Dulbecco’s modified Eagle’s medium
EDTA: Ethylenediamine tetraacetic acid
EGF: Epidermal growth factor
EMT: Epithelial–mesenchymal transition
FACS: Fluorescence-activated cell sorting
FBS: Fetal bovine serum
FGF: Fibroblast growth factor
FTC: Fumitremorgin C
HSP: Heat shock protein
MACS: Magnetic-activated cell sorting
MEM: Minimum essential medium
miRNA: Micro RNA
MSC: Mesenchymal stem cell
NCS: Neonatal calf serum
PBS: Phosphate-buffered saline
PI: Propidium iodide
RCC: Renal cell carcinoma
Rh123: Rhodamine 123
SFDM: Serum-free defined media
siRNA: Small interfering RNA
SP: Side population
SR-FTIR: Synchrotron radiation–Fourier transform infrared
tAPC: Tubular adult renal progenitor cell
TCGA KIRC: The Cancer Genome Atlas’ Kidney Renal Clear Cell Carcinoma
TGF: Transforming growth factor
TIC: Tumor initiating cell
